# Vision-Based Damage Detection for One-Fixed-End Structures Based on Aligned Marker Space and Decision Fusion

**DOI:** 10.3390/s22249820

**Published:** 2022-12-14

**Authors:** Ziemowit Dworakowski, Pawel Zdziebko, Kajetan Dziedziech, Krzysztof Holak

**Affiliations:** Department of Robotics and Mechatronics, AGH University of Science and Technology, 30-059 Kraków, Poland

**Keywords:** vision system, deflection, damage detection, decision fusion

## Abstract

It is possible to detect damage in structures based only on vision-system-based assessment of their deformation shape under load. There is, however, a gap between available methods designed to detect damage in beam-like structures and engineering needs for monitoring structures of many different shapes. In this article, a new Aligned Marker Space method of morphing vision data is introduced. The method allows damage detection of any engineering object with one fixed support as if it were a cantilever beam. The paper also presents a new fusion technique to combine the results of several damage-detection methods for an increase in accuracy and sensitivity. The methods are tested based on numerical simulation of various structures, a blender-based simulation, and a set of practical experiments in which crane structures are subjected to damage of different sizes and locations. The optimization of damage detection methods’ metaparemeters is performed using an evolutionary algorithm designed to find the Pareto front of the solutions. The assessment of the influence of different factors, like camera position, damage position, or repetition of the experiment, is provided.

## 1. Introduction

Vision systems are very popular monitoring methods due to their full-field measurement and noncontact nature, and for these reasons are often used in many areas of engineering [1,2,3,4]. In the field of vision-based damage detection, calculation and analysis of Deflection Shape (DS) often plays a major role. Internal cracks propagating inside a structure may locally lower the structure’s stiffness, which can be seen as a dent or a break on the DS under load. The most straightforward approach to detect such a change is to acquire the shape of deflection in a known, intact state and then use it as a reference (baseline) to compare it with shapes obtained in an unknown structural state. The DS is usually a finite set of displacements measured in respective points of the structure, usually in attached vision markers. The measurement is often done with means of Digital Image Correlation [5,6,7]

If the environmental conditions, including load causing the deflection, are similar, the differences in acquired DSs are an indication of damage. These differences are usually analyzed through calculation of Deflection Difference (DD) signal, being a subtraction of reference DS and potentially damaged DS. The DS-based approaches have several limitations, including necessity to provide a good point of reference for reliable mounting of a sensor or compensation of its location difference, and they work in situations when the damage developing in the structure affects structural behavior locally and in the plane that is observed by the vision sensor. For this reason, they can not be used e.g., for corrosion detection—for which other methods, including deep learning are of great interest [8]. However, low cost of DS measurements and their ability to acquire a full-field of DS encompassing the whole structure within one measurement triggered development of numerous systems and methods aimed to detect local damage by baseline-based analysis of the DS.

Various algorithms based on the analysis of irregularities in the 1D displacement, deflection, or curvature signals have been developed. Gauthier et al. [9] experimentally validated an application of second and higher-order derivatives of deflection for crack detection in beam-like structures. Li et al. [10] applied fractal dimension analysis for damage detection in beams of uniform cross-section. Jing et al. [11] used the roughness method to detect damage as an irregularity of the displacement signal’s rough part. RaghuPrada et al. [12] proposed detection of symmetry loss in the deflection curve as a damage presence indicator. There are numerous methods based on strain energy analysis. For example, Seyedpoor et al. [13] presented damage indicator based on changes in the strain energy computed from noisy static measurement data. Guo [14] combined strain energy and evidence theory. Strain energy analysis was applied to quantify the extent of damage, while evidence theory was used to specify its location. Many authors developed damage indices based on wavelet transform (WT) of the deflection curve. In the earliest works, the method has been limited to detection of a single crack in beam-like structures of uniform cross-section [15,16]. Damage is located based on the analysis of singularities in the transformed deflection profile. Wavelet transform maxima are taken as damage indicators. Zhu [17] applied WT to analyze crack presence in functionally graded beams. Loutridis et al. [18] increased the damage identification capability of continuous wavelets to double-cracked beams. Janeliukstis et al. [19], and Ma et al. [20] applied WT to identify multiple damages in beam-like structures.

Abdo [21] applied grey relation theory for damage localization. Changes in the displacement curvature and the grey relation coefficient have been used to compute crack location and estimate its severity. Dworakowski et al. [22] applied the line segment method to detect the location of damage in cantilever structures and quantify its extend based on image data. Homography mapping was introduced to reduce the influence of perspective distortion on measurement. Yang et al. [23] proposed structural damage location and quantification based on flexibility disassembly technique and the concept of damage localization vector. However, the developed approach requires a precise FEM model of the undamaged structure as a computation baseline. Wang et al. [24] presented a method that combines static deflection and natural frequencies information to identify damage. Its severity is evaluated using iterative quadratic programming. Le et al. [25] developed a method of location and quantification of damage by static deflection change function, with a linear relationship to a damage severity derivative. A new damage indicator based on the damage-severity-consistency concept was proposed.

One of the most significant drawbacks of the crack localization methods based on curvature analysis is the sensitivity to noise in the measurement data. Bakhtiari-Nejad et al. [26] proposed an optimization criterion to detect damage from noisy data by minimizing a difference between load vectors, computed before and after the appearance of damage. Hensman et al. [27] proposed replacing derivative computation with Gaussian processes to avoid numerical differentiation problems.

An interesting emerging group of damage detection methods is the approach utilizing moving loads for damage identification. It found an application in the deflection analysis of bridges under operation. O’Brien et al. [28] proposed a Moving Force Identification algorithm to detect bridge damage. Two damage indicators and their combination have been proposed. Mouseavi et al. [29] presented a damage detection method based on the integral of crack-induced deflection of the beam under quasi-static moving load. The method was evaluated on a simple supported beam structure. The same authors, in a more recent paper [30], proposed a baseline-free damage detection method for beam structures subjected to a moving load. New damage indicator formulas have been developed for different boundary conditions of the beam. A static condensation scheme was proposed to reduce the amount of required information.

In all these methods, usually, a simple linear cantilever beam is used as a proof-of-concept. It is caused by the fact that geometry changes alter the deflection and, in many cases, render it difficult to analyze. Additional assumptions include constant stiffness over the whole structure under investigation and the uniform marker spacing—as changes in each of these parameters are often reflected in breaks or dents in the DD as well, causing false-positive damage indications.

In this paper, a novel Aligned Marker Space (AMS) method of transforming the deflection data is proposed, such that it can be used to build easy-to-analyze DSs even if the markers are not uniformly spaced, the structure’s stiffness is not constant, and the structure’s geometry is complex. These DSs are analyzed using a variety of deflection-based damage detection methods, namely: a Second Derivative (SD) method, a Wavelet (WV) method, a Line Difference (LD) method, a Line Segment (LS) method and, finally, a Field Fusion (FF) method that combines them all into one decision engine. A Blender-based simulation is introduced as a means of methods’ configuration and evaluation and to facilitate the practical implementation of the approach for new structures. Then, the configured methods are tested in a series of experiments using a crane structure of varying stiffness that is damaged in several locations.

The main novelty of the article is based on three claims:

1 The AMS method allows us to use beam-based damage detection and localization methods to various non-beam configurations (cranes, curved beams, etc.)
2 Decision fusion of various damage detection methods increases the reliability of the solution. The fusion can be performed both in the sub-method space and measurement space by aggregating results from different methods and from different measurements.
3 It is possible to optimize meta-parameter values for the FF method based on beam data and then, with the help of the AMS, use it to detect damage in a new structure.

The remainder of this article is organized as follows: Section 2 introduces the AMS method used in the article, and provides initial numerical verification of its capabilities. Section 3 itroduces deflection-difference-based damage detection methods for cantilever beams. Section 4 introduces evolutionary optimization routines used for configuration of field fusion methods. Section 5 provides Finite Element Model (FEM) and Blender simulation-based verification of the damage-detecion methodology. Section 6 provides results of a practical experimental evaluation of the methods. Finally, Section 7 summarizes and concludes the paper.

## 2. Aligned Marker Space

One of the main limitations of the existing deflection-based damage detection methods is the requirement of the structure to be cantilever-like. This can be solved using the AMS approach.

### 2.1. The Method

The algorithm’s starting point is a matrix of point coordinates (either in 2D or in 3D). The order of points in the matrix is used to build a new, non-euclidean transformation of measurement space into AMS. The new *x*-axis is built from coordinates of points so that each consecutive point is placed on the axis, and its distance to its neighbor to the left (previously considered point) is equal to the euclidean distance between these two points in measurement space. The method thus straightensa curvature of the object to a straight line. Future baseline-based displacements will be recorded perpendicularly to this line. Algorithm 1 for obtaining a vector of new coordinates is as follows:
**Algorithm 1**: AMS transformation.1:*set* pointer *p* to 02:*set*k=23:**while**k<= Number of measurement points **do**4:   calculate euclidean distance *d* between measurement points *k* and k−15:   Store coordinate (p+d) in matrix *C*6:   *set* pointer *p* to p+d7:   Increment *k*8:**end while**9:**return** Matrix *C* with new coordinates of points in AMS

**Figure 1:**
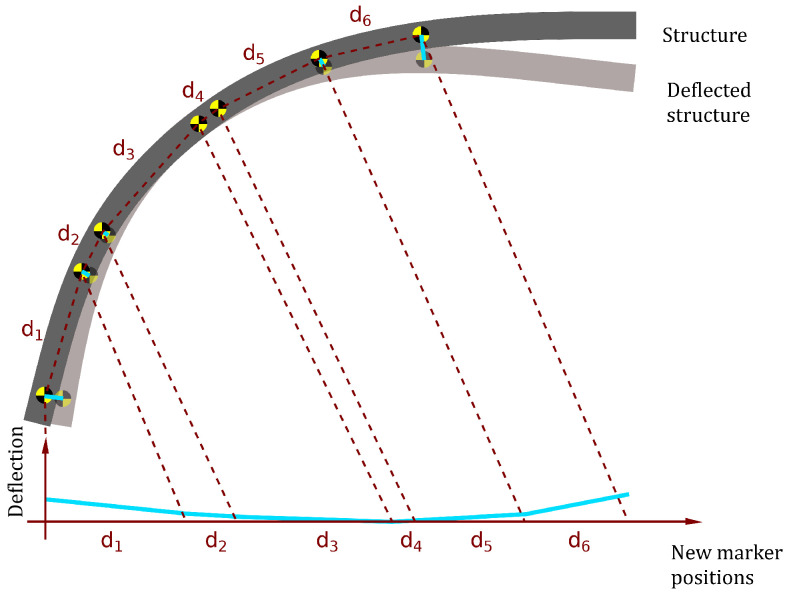
Illustration of the AMS method in practice. The non-beam structure is deflected (e.g., due to damage presence). The differences in deflection for the respective markers are mapped to a new set of coordinates. The Euclidean distances between markers are preserved.

As a result, AMS is defined by adding a new separate spatial dimension to measurement space for organizing the points and then shrinking all three initial spatial dimensions into one for storing displacement of the point from its initial location. It is worth noting that instead of shrinking the 3D space into a 1D displacement axis, it is possible to calculate displacements separately for all three axes and storing all of them in a 4D AMS: the first dimension for the organization of points followed by three dimensions for displacements in 3D. An illustration of the process is provided in Figure 1.

### 2.2. FEM-Based Concept Validation

**Figure 2:**
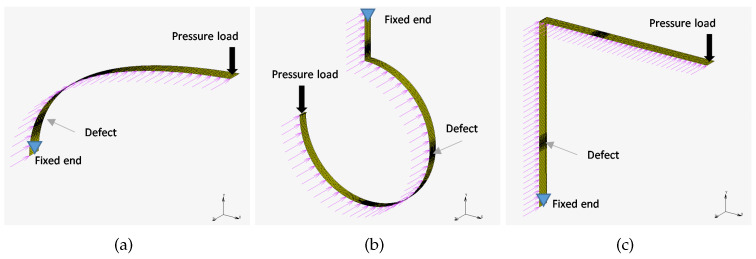
Three structures used for initial AMS verification: (**a**) a curved beam, (**b**) a hook, and (**c**) an L-shaped crane.

Three numerical models were developed and computed to illustrate how the method allows for straightening of various structures and reduction of markers under observation. The FEM was adopted to calculate the position of points on investigated structures—as depicted in Figure 2.

The numerical models of the following shapes were taken into consideration:

- Curved beam—see Figure 2a
- Hook—see Figure 2b
- L-shape crane—see Figure 2c

**Figure 3:**
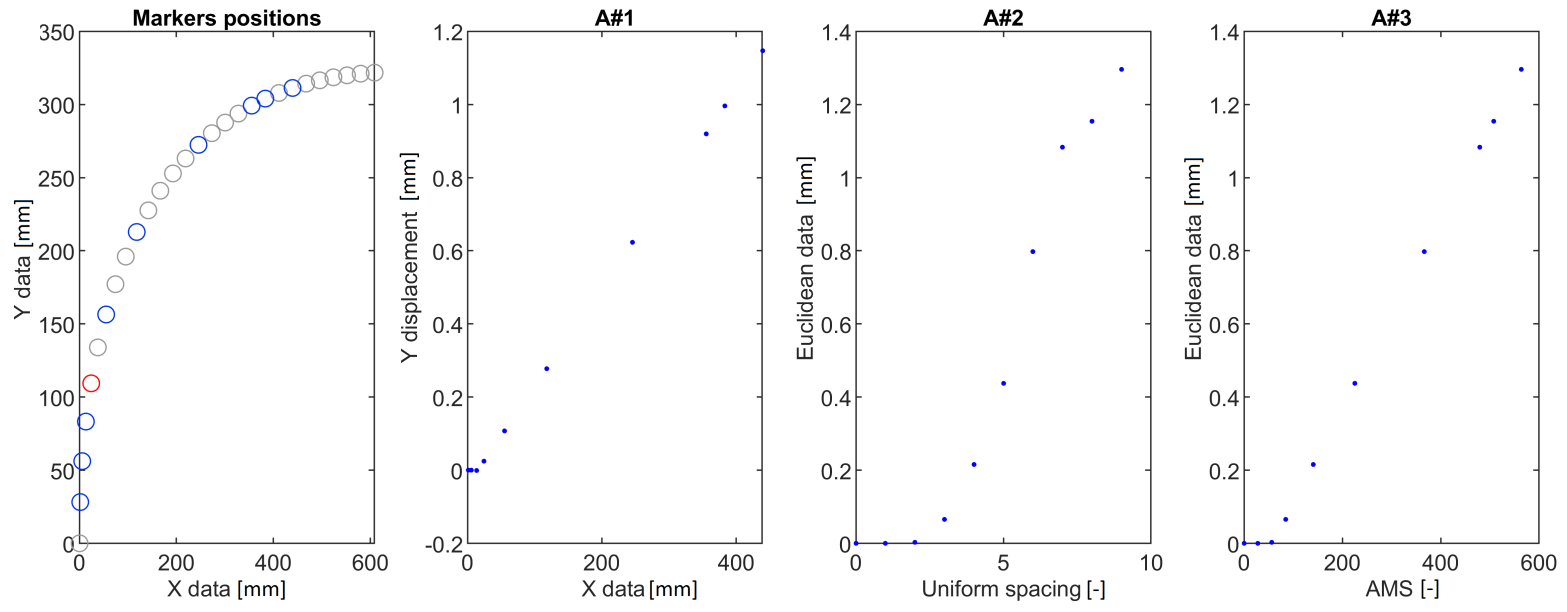
DDs for a curved beam structure calculated using the three tested approaches. Blue circles denote points used for evaluation. Grey circles denote points randomly excluded from measurement. The red circle denotes the damage location.

The Finite Emelent (FE) models were formulated using the Altair HyperMesh pre-processor and solved in MSC.Marc solver. The 2D element types were chosen because the thickness of modeled components is significantly lower when compared to the other dimensions. The boundary conditions and defects introduced in each model are presented in Figure 3. Pink arrows depict points (markers) on structures that are analyzed using the AMS approach. The numerical analysis type is linear static, and the magnitude of pressure applied to the structures was chosen to cause 5% rate of deflection to the original diameter (in case of the hook structure), or 5% rate of deflection to the original length (in case of the curved-beam and L-shape crane structures). As a result of the numerical simulations, actual positions of markers on structures were computed for cases with and without defects. This data is then used for DDs calculation.

The calculated DDs were analyzed using the following approaches:

- A#1 Calculation of a vertical displacement difference as a function of marker position along horizontal axis (a standard approach).
- A#2 Calculation of the euclidean displacement difference as a function of marker number.
- A#3 Mapping the displacements using AMS.

**Figure 4:**
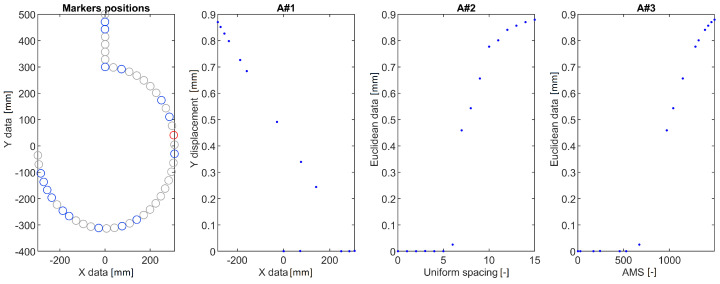
DDs for a hook structure calculated using the three tested approaches. Blue circles denote points used for evaluation. Grey circles denote points randomly excluded from measurement. The red circle denotes the damage location.

**Figure 5:**
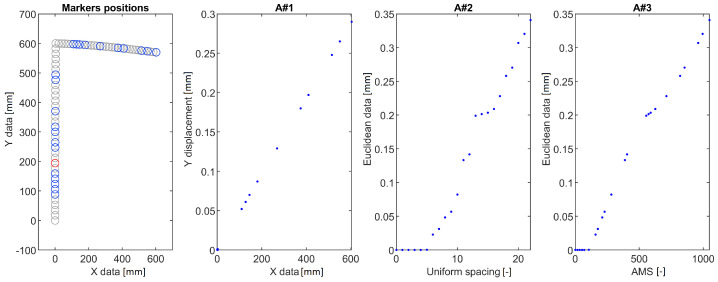
DDs for an L-shaped crane structure calculated using the three tested approaches. Blue circles denote points used for evaluation. Grey circles denote points randomly excluded from measurement. The red circle denotes the damage location.

The DDs obtained for the three structures and the proposed methods are shown in Figure 3, Figure 4 and Figure 5. In each of the provided figures, the leftmost panel shows the actual positions in X-Y coordinates of all the points taken into consideration. Two-thirds of these points were randomly excluded from further calculations to simulate a bad case of a condition in which vision markers are either placed non-uniformly or rendered partially useless due to, e.g., errors in tracking. Each figure’s remainder is devoted to a visualization of the DD using the three approaches. The goal here is to obtain a plot that can be used as an input for damage detection algorithms: It should have values close to 0 in the area between fixed end and damage and a clear linear rise in the section between damage and a free end of a structure.

The A#1 approach was provided here only for the sake of consistency—as visualization of vertical displacement along a horizontal axis is efficient only for beam-like structures. Here it failed to provide meaningful information for all the cases investigated. It either provides a complex plot in which it is hard to denote where the damage is located (Figure 4) or shrinks all the points of similar X coordinate to just one point (Figure 5). The A#2 approach fares better here as the damage location is always a beginning of an evident rise but is vulnerable to marker density changes. These changes are reflected as changes in DD steepness, which could trigger false positive detections using methods that consider the local shape of the DD. Finally, the proposed AMS approach provides results similar to the A#2 one, with the rise being much more linear and easier for the damage detection algorithms to work with.

## 3. Damage Detection Methods

### 3.1. Second Derivative Method

**Figure 6:**
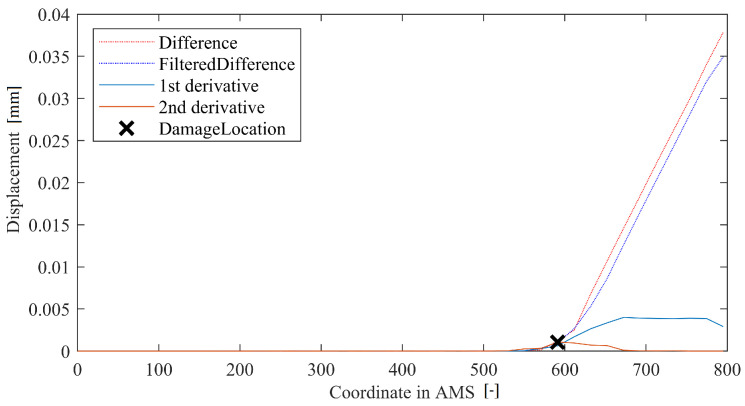
Example of operation of the SD method.

The method based on the article by Dworakowski et al. [22] requires the calculation of the second derivative of a DD. The maximum absolute value of that signal indicates possible damage location. The rationale behind this is as follows: The damage usually causes the line of deflection to change its angle rapidly. The first derivative thus changes its value significantly. As a result, the second (double) derivative form a clear peak in the respective area. In order for the method to work, a filtration step is necessary to suppress measurement noise. Thus, the method has one meta-parameter: a length of filter mask (usually set up roughly at 5% of structure’s length). The filter mask size determines the noise-filtering capabilities of the method. The bigger it is, the less it is affected by the measurement noise. However, its size proportionally reduces the method’s spatial resolution and sensitivity to small damage. A method in-operation for an ideal case of beam data works as depicted in Figure 6.

### 3.2. Line Segment Method

**Figure 7:**
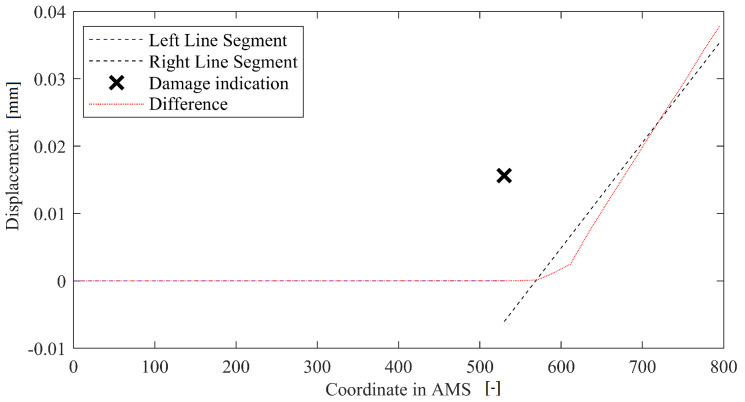
Example of operation of the LS method.

The method is again based on previous work by Dworakowski et al. [22], and it consists of fitting two line segments into the deflection curve to minimize the mean squared error between segments and the measurement points. The angle between line segments with the best fit is a damage indicator; the point at which the segments connect is a damage location. When there is much noise present in the data and the detection is baseline-based, additional assumptions can be made: one of the segments must be horizontal. A method in-operation works as depicted in Figure 7. The method has one meta-parameter: offset that determines the minimal length of the possible line segment. The offset should be shorter than the minimal distance between the expected damage location and the monitored beam’s end. However, the shorter it is, the more noise-sensitive the method gets. The parameter was arbitrarily set up as 10% of the beam’s length.

### 3.3. Line Difference Method

**Figure 8:**
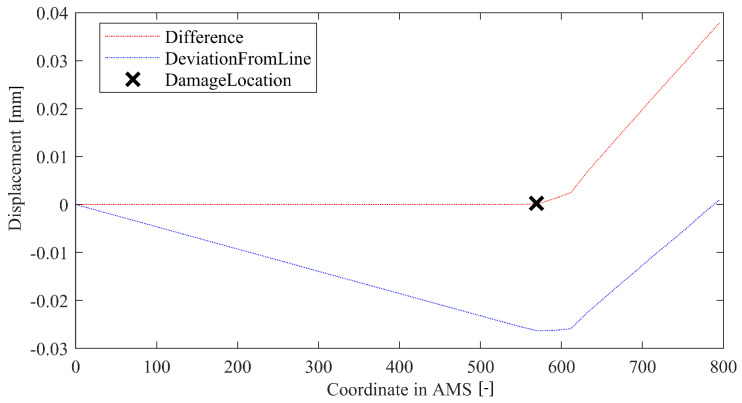
Example of operation of the LD method.

The basic idea of the LD method involves calculating a coefficients for a line connecting both ends of a DD curve. This line is then subtracted from data resulting in a deviation from a straight line. The maximum of this deviation is treated as a damage indicator, while its location is marked as the damage location. A method in-operation is depicted in the Figure 8. It is a straightforward method that thrives on the assumption that the structure’s geometry is straight. Therefore, it can cooperate well with the AMS approach. The method has no metaparameters.

### 3.4. Wavelet Method

**Figure 9:**
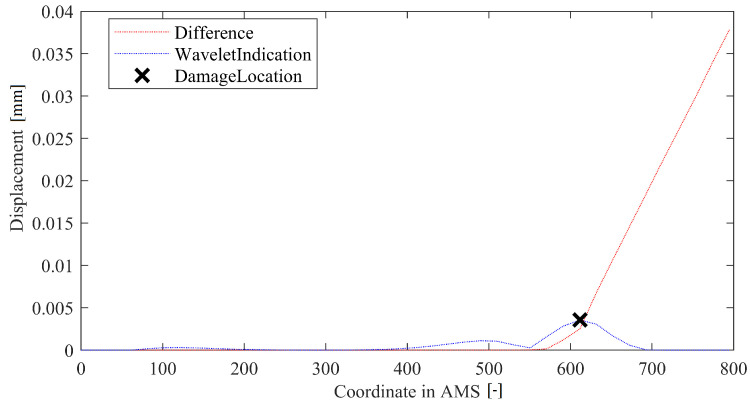
Example of operation of the WV method.

The WV method involves calculating a wavelet transform of results of a LD method. Maximum of the absolute value of scalogram averaged over the selected range of scales is treated as damage indication (with the location being equal to the location of the maximum). A method in-operation is depicted in the Figure 9. The method has three metaparameters: Offset and two (upper and lower) wavelet ranges. The offset plays a similar role as in the LS method; that is, it prevents the method from being over-sensitive at the deflection curve’s edges. Here, it was set up similarly as before, at 10% of the DS’s length. The Wavelet range is a vector that determines a range of scales in which the wavelet signal is added together. Here, it was used to filter out the highest scales (as they are sensitive to damage in general but reduce localization capabilities) and lower scales (because they are sensitive to noise rather than a general change of the deflection curve’s direction).

### 3.5. Field Fusion Method

**Figure 10:**
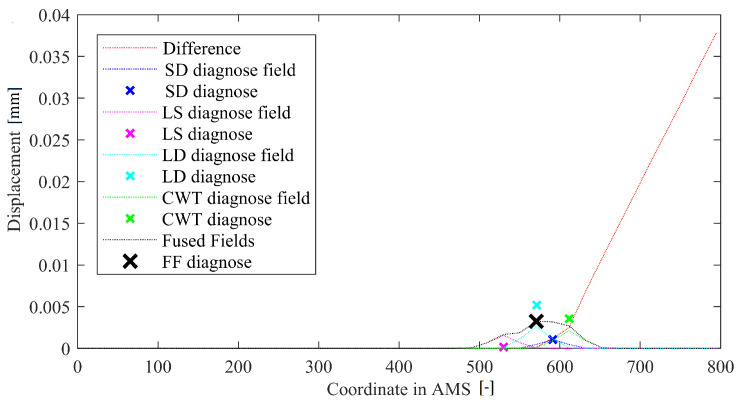
Example of operation of a FF method.

Finally, the last method (FF) fuses all of the above-described components into one decision. The idea involves calculating positions and values for damage indications from respective methods and then using these to generate Gaussians of signal in AMS. For each component, the Gaussian has an amplitude equal to damage indication multiplied by a weight and pre-defined width—centered at the source method’s damage location. The method thus has 8 metaparameters which are denoted as weights $W_{LD}$, $W_{LS}$, $W_{SD}$, $W_{WT}$ and sigmas $S_{LD}$, $S_{LS}$, $S_{SD}$, $S_{WT}$. There is also the decision threshold $T_{FF}$, but since the eight basic parameters govern the outcome, the threshold can be set to 1. These meta-parameters from respective groups can either be set at equal values (if one assumes that all the four above-described methods have equal efficiency) or optimized based on preliminary experiments. A method in-operation is depicted in Figure 10.

## 4. Method Configuration

To optimize the internal weights of the FF data obtained in the previous experiment was used [22]. These data obtained in a series of static experiments in which cantilever beams were loaded and damaged consisted of 241 damage scenarios with variable severity divided among 25 different locations spanning over the 350 mm range. The area under monitoring, i.e., the area in which damage was searched for, equaled 600 mm. The optimization here has two goals (i.e., it is a two-criterion problem): it is required to minimize location error of the methods and, at the same time, maintain a low number of “misses”—so damaged cases denoted as “intact”.

As a solution for optimization of FF metaparameters a Evolutionary Algorithm (EA) was designed. The two-criterion problem was solved using the Pareto optimization approach: Solutions were arranged in order of raising the number of misses and decreasing accuracy (distance to the actual damage). In this two-criterion space, a Pareto front was calculated. Individuals located at the front were passed without modification to the next generation. The remaining space in the offspring population was filled by random mutations of the individuals located at the front. The selective pressure was relatively high and variable, as only a few individuals were usually located at the front. The initial population was generated so that weights and radii starting range was (0; 200). No algorithm meta-parameter optimization was performed because the problem was relatively easy with consecutive runs of the code resulting in relatively similar solutions. Parameters of EA are provided in the Table 1.

**Figure 11:**
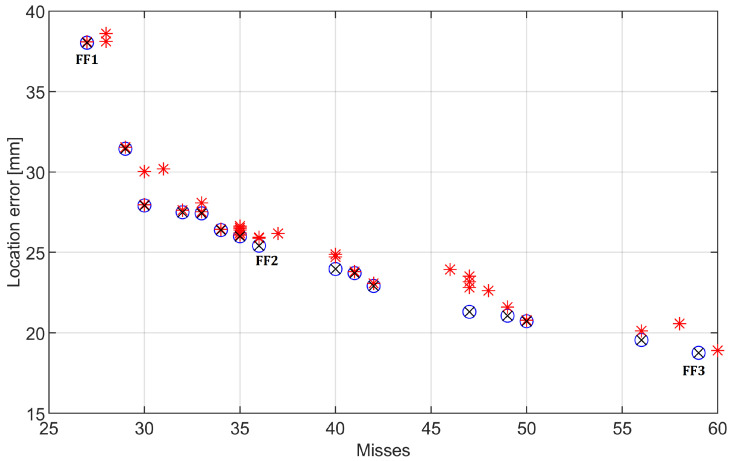
Result of the optimization process. Red asterisks refer to results of all individuals from the final population. Pareto front is denoted as circles. Three selected optimization results are denoted with ‘FF1’ to ‘FF3’ symbols.

In Figure 11 the final result is depicted. The location error should be considered concerning the area under monitoring, so the 80 mm damage location error would mean approximately 12% of the beam’s length. It can be seen that there is a clear trade-off between the number of misses and the location error. Three cases were selected as ’the most interesting’: FF1 case optimizes the number of misses (i.e., the method should provide damage detection as often as possible, at the cost of location accuracy), FF2 provides a relatively low number of misses while maintaining a high location accuracy), and FF3 optimizes location error at the cost of a number of misses. These results, along with the optimized weights, are provided in Table 2. The detection error is calculated for all 241 cases investigated (note that some of the damage scenarios have a negligible effect on the beam—as presented in the previous article [22], the location error is calculated with respect to the length of the area under monitoring (600 mm)). The optimized weights between cases FF1 and FF3 tend to get smaller—which is expected and caused by the allowed reduction of misses. In other words, if the method should provide the lowest location error possible, it would fire rarely and only if the damage location indication is clear and sharp. To this end, the weights are smaller, so they would much less frequently exceed the threshold. The weights cannot be viewed as a direct indication of ’which method is deemed better’ by the optimization algorithm as they are multipliers for the basic methods’ indications. An interesting observation is that the WV method was often ’turned off’ in the optimization: the respective weights were set to 0. This was true for 5 out of 10 cases present in the final Pareto front. A possible explanation is that the method tends to operate similarly to DD but is weaker (i.e., more sensitive to noise) and therefore often fails to contribute to a fusion method.

## 5. Simulation-Based Evaluation

For the first-stage evaluation presented in this paper, a numerical setup that is based on the results of FE Analysis (FEA) and a computer graphics program (Blender) was developed. The algorithm’s result is an image rendered in a graphic program, which presents the examined structure. It is synthetically produced based on the deformation determined by the FEM solver. Numerical models allow us to significantly reduce the time for vision data creation compared to real-life experiments.

**Figure 12:**
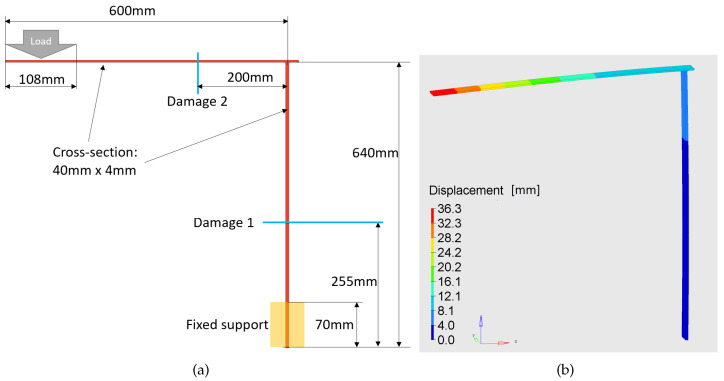
The L-shape crane structure under investigation: (**a**) scheme with dimensions, and (**b**) displacements computed by the FEA.

The developed algorithm workflow is as follows: nodal displacements of the structure under study are determined as a result of the FEM analysis. For the evaluation presented in this paper, the L-shape crane model was created. Figure 12a shows a scheme of the structure under investigation, and Figure 12b presents the deformed FEM model. Two cases were tested: one with damage located at a horizontal bar and one with damage located at a vertical bar.

**Figure 13:**
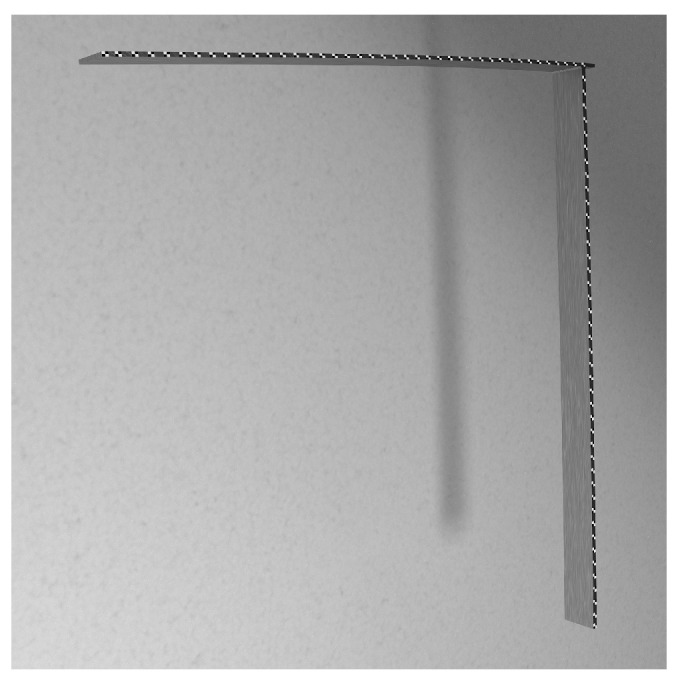
Rendered image of the L-shape crane structure under load.

The deformed mesh of the model is exported to the *.STL format, and then loaded in the Blender graphic program. Materials are next assigned to the appropriate parts of the mesh, characterized by a given color, texture, and the means of light diffusion. Additionally, the camera system settings are configured: lighting, camera position and orientation, camera sensor parameters, and the focal length of the lens. The camera system settings modeled in Blender are as follows: camera resolution: 5616 × 3744 pixel, focal length: 30 and 32 mm, sensor size: 24 × 36 mm—those parameters correspond to full-frame DSLR (digital single-lens reflex camera) Canon EOS 5D Mark II. Image rendered using the developed setup is shown in Figure 13.

The procedure was repeated for the loaded state with damage and without damage. The intact scenario was used for the reference deflection state calculation. The obtained photos are input for further processing using the image processing and damage detection algorithms.

**Figure 14:**
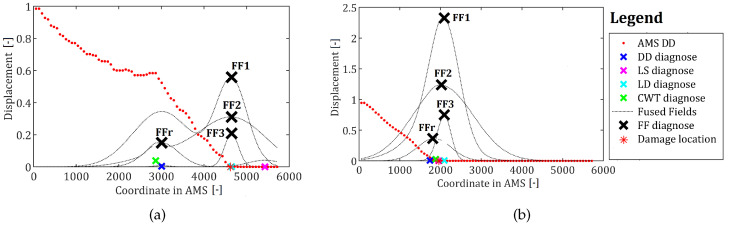
The results of all the damage detection methods for (**a**) damage located in a vertical bar and (**b**) damage located in a horizontal bar. The coordinate in AMS axis refers to the markers positions’ in a transformed space. The beginning of mapping started at a free end of the crane.

The resulting deflection difference curves for both locations of damage, mapped into AMS space and subjected to diagnosis using all the tested solutions, are depicted with the respective damage locations in Figure 14. The points are ordered starting from a free end of a crane; thus, the point at the intersection of the horizontal and vertical parts is located around the middle, and the point at the fixed end is the final point of the series. The coordinates for these points are around 0, 3000, and 6000, respectively. In both cases, the damage is detected correctly, and thus the mapping into AMS is working as intended. It is worth noting, however, that damage present in a vertical part of a crane rendered the defected shape to be bent around the middle, causing false indications of the DD and WV methods, rendering the FFr configuration of the method to be incorrect. The remaining methods operated properly. For the damage located at the horizontal beam, all the methods worked as intended. It is also worth noting that the final damage indication is almost four times as high for the horizontal beam than for the vertical one.

## 6. Experimental Evaluation

### 6.1. Experimental Setup

The second stage of the evaluation was experimental. The research was carried out using the same L-shape crane structure as shown in Figure 12a. The two samples were produced by welding two steel beams with the given dimensions. The boundary conditions in experiments were consistent with those used in the simulation approach. The experimental cases were organized according to the Table 3. Damage location 1 refers to damage located at a vertical part of the crane. Damage location 2 refers to damage located at the horizontal part of the crane. The consecutive pairs of cases (that is, cases C#1 and C#2, C#3 and C#4 and so on) were acquired simultaneously, using two independently placed cameras, with Camera 1 denoting the camera with optical axis was perpendicular to the crane surface. Camera 2 was located at an angle. Other than that, cases are ordered by the time of their acquisition. Note that the order of damage introduction was reversed for the second specimen: the horizontal beam was damaged before the vertical one.

**Figure 15:**
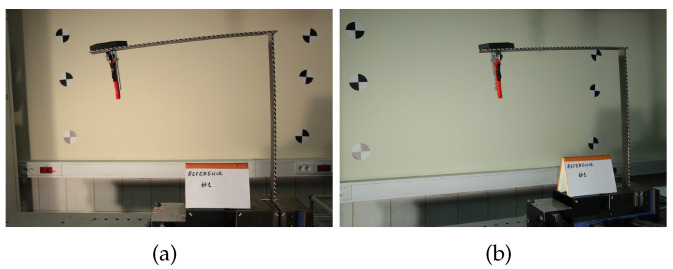
L-shape crane images captures in experiments: (**a**) front camera view, (**b**) side-front camera view.

**Figure 16:**
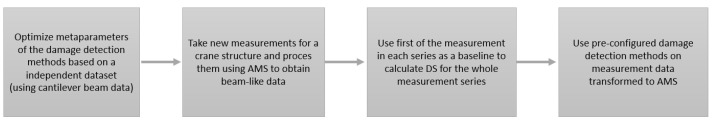
Flowchart of the AMS method.

Each experimental case involved introducing ten damage sizes starting from 2 mm and going up to roughly 20 mm. After each damage introduction, about 1 min time interval was used in order to dampen vibrations. Then, at least 20 images were captured using each camera. Sample images captured in the experimental tests are shown in Figure 15. A general scheme of data processing, higlighting the fact that sources for method evaluation and configuration are different, is depicted in Figure 16.

### 6.2. Example of Results from One Experimental Case

**Figure 17:**
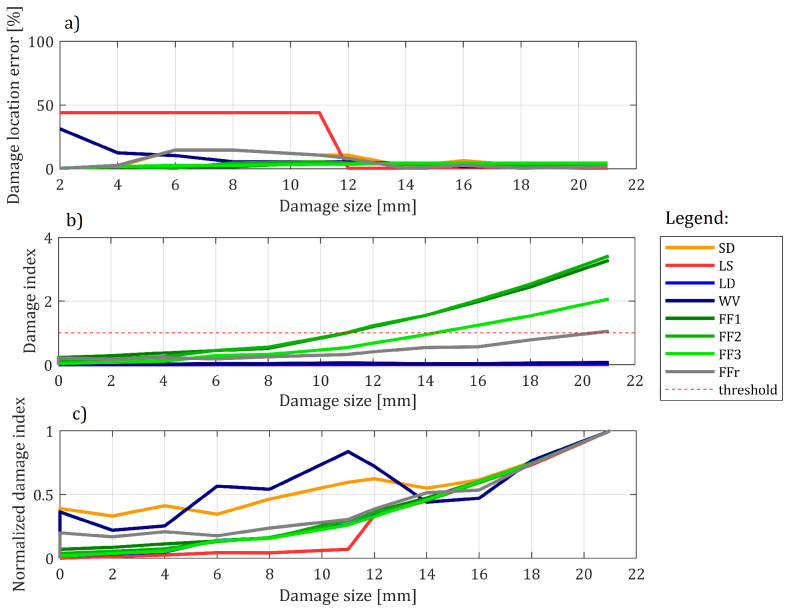
The results obtained for testing the 8 damage detection methods for one particular setup of the experiment (C#3): (**a**) Relative damage location error; (**b**) damage indication and (**c**) damage indication normalized with respect to the maximum for the given method.

A result for one particular setup (that is: for one camera, one damage location and one of the two specimens) is shown in Figure 17. Apart from the source methods (that is: the DD, LS, LD and WV), four fusion solutions are also evaluated. Three of them (namely: FF1, FF2 and FF3) correspond to three chosen optimized setups presented in Section 4. The final FFr solution is taken from a previous paper [22], so consist only of fusion of DD and LS methods. The weights are optimized as in Section 4 and are equal to 40 and 350 respectively for DD and LS counterparts.

It can easily be seen that that all the methods’ damage indications tend to correlate with damage size. It is also clear that the higher the damage is, the more accurate the methods are in its location. Note that in Figure 17c, damage indications are normalized, so the maximum indication reported by the method is equal to 1; all the remaining values are scaled with respect to these maxima. The reason for this action lies in providing a possibility of comparison of all the methods. Ideally, the indication of damage should be as low as possible for the ’0’ size of the damage and should gradually increase for the latter cases. It can be seen that, while the fusion scenarios generally follow the expected path, some of the underlying methods differ significantly from the expectation. Both DD and WV methods tend to show relatively high damage indications for an intact state of the structure—thus causing the necessity for high detection threshold setup if they were to be used on their own. In Figure 17b, the non-normalized plot allows for evaluating the damage detection efficiency of the fusion methods with respect to the preoptimized thresholds. Damage is detected once a damage index exceeds 1. As expected, the FF1 and FF2 configurations are more sensitive to damage than FF3. The former detect damage at 12 mm, while the latter succeeds at 16 mm. Finally, the FFr method allows for the detection of damage only at maximum size.

In the remainder of the paper, the damage detection capabilities will be evaluated in a non-normalized manner to allow for threshold setup based on preoptimization routine verification. Due to that, the base methods (that is: the DD, LS, LD and WV indications) will be omitted to maintain clarity and because there is not enough data in the testing subset to calculate thresholds for each of the underlying methods in an unbiased manner.

### 6.3. Influence of the Various Experimental Conditions

In this subsection, the camera location’s influence (located at a straight angle or tilted), specimen used (so repeatability of the experiment), and influence of damage location are investigated.

#### 6.3.1. Camera Influence

**Figure 18:**
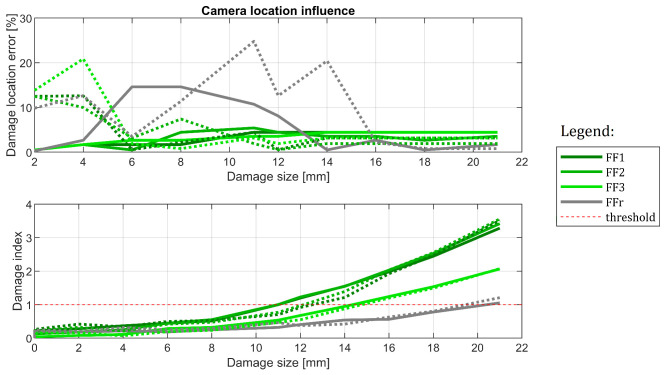
The results obtained for cases C#3 and C#4, that is for the same specimen and damage case, but with two different camera locations. Results from Camera 1 are marked in dashed lines.

Cases C#3 and C#4 were used to evaluate how camera location influences the results. The results arranged similarly as in the previous example are provided in Figure 18. It appears that this source of variability is relatively the smallest—with damage indices following the same paths for both sources of data. Regarding damage location capabilities, detection based on Camera 1 tends to be slightly more accurate in the latter part of the experiment—especially for FF1 to FF3 setups. FFr produces some significant location errors, but this can be treated as noise because damage indication suggests that the method did not find any damage before 16–18 mm severity. The conclusion here is that as long as the camera is placed roughly in front of the monitored specimen, the obtained results should not differ much. It is worth mentioning that no rectification procedure was used here; the AMS method is solely responsible for the good results.

#### 6.3.2. Influence of the Specimen

**Figure 19:**
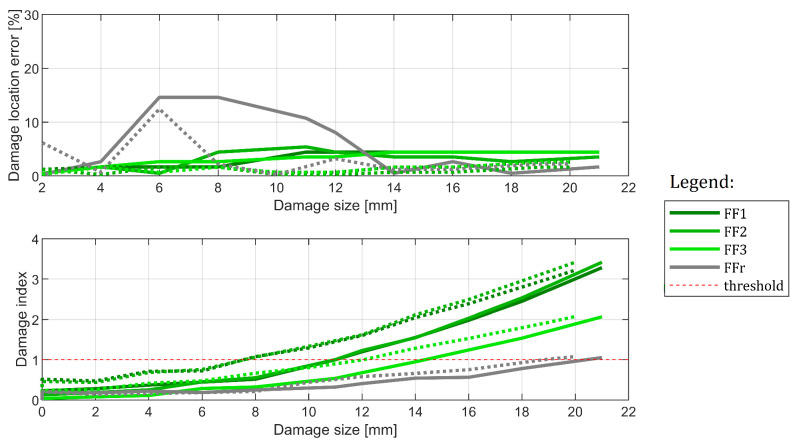
The results obtained for cases C#3 and C#5, that is for the same camera and damage location, but for two different specimens. Results from specimen 2 are marked in dashed lines.

In this section, cases C#3 and C#5 are compared with each other, as presented in Figure 19. The goal here is to evaluate specimen-based variability. This does not strictly cover the experiment’s repeatability because damages are introduced in a different order for both specimens. That is: for C#5, the evaluated case contains one damage only, while for C#3, it is a pre-damaged specimen with one damage already existing in the vertical beam. While the method should generally ignore it, as the pre-damaged state serves as a baseline in this experiment, this damage may influence how the structure reacts to the second damage. In this experiment, the damage location capabilities of the methods are again similar. While these obtained from the second specimen are slightly more accurate, the difference is still roughly within 2% of the structure’s length. Regarding the damage detection capabilities, the results are more significant: for the second specimen, the damage is detected usually one or two growth stages earlier. The only exception is the FFr method, for which this difference is much less visible. The conclusion here is that, while the methods’ damage location capabilities do not depend on the specimen used and are easily repeatable, the point at which damage indication exceeds the detection threshold might differ significantly.

#### 6.3.3. Influence of the Damage Location

**Figure 20:**
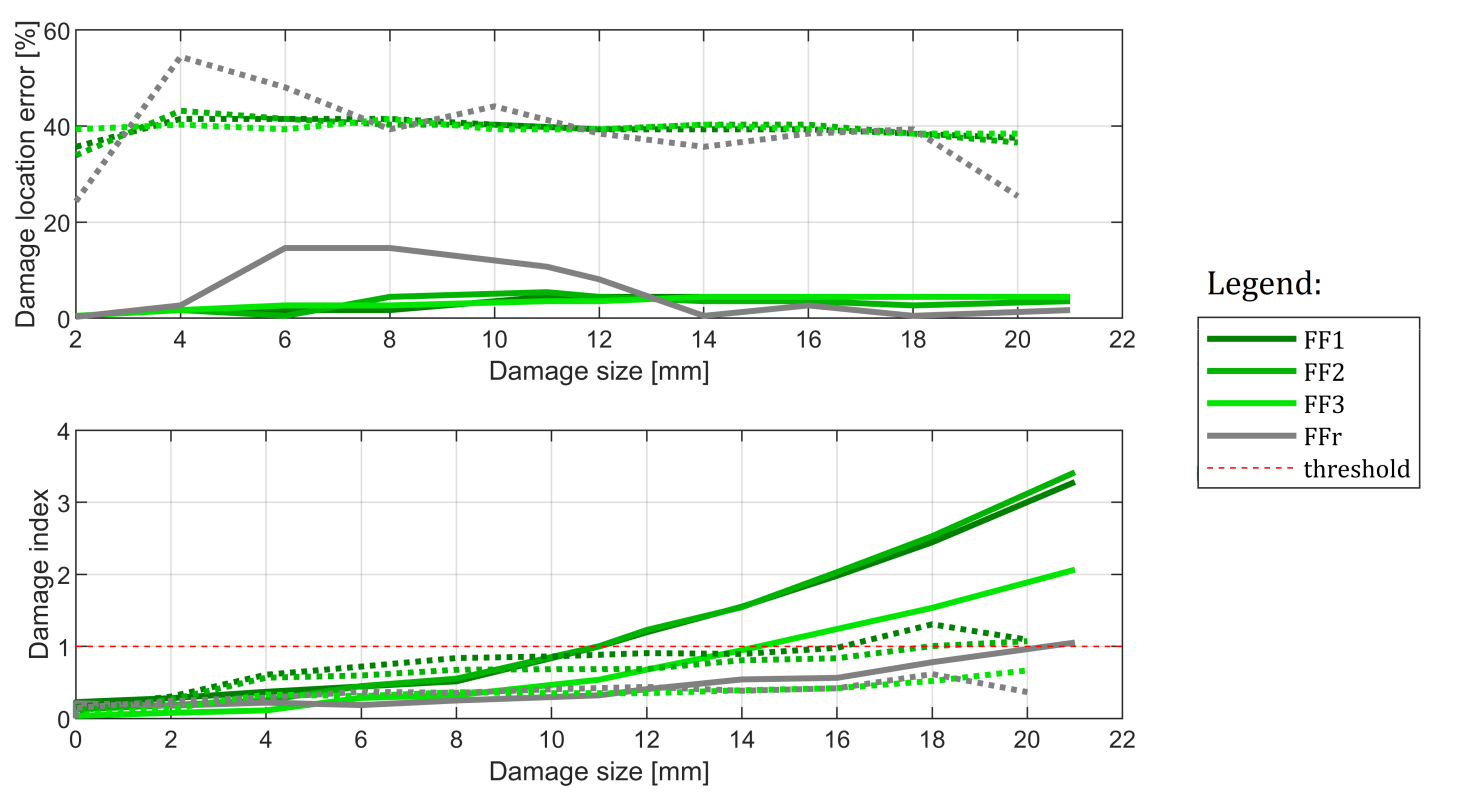
The results obtained for cases C#1 and C#3, that is for the same camera and specimen, but for two different damage locations. Damage location 2 is marked in dashed lines.

**Figure 21:**
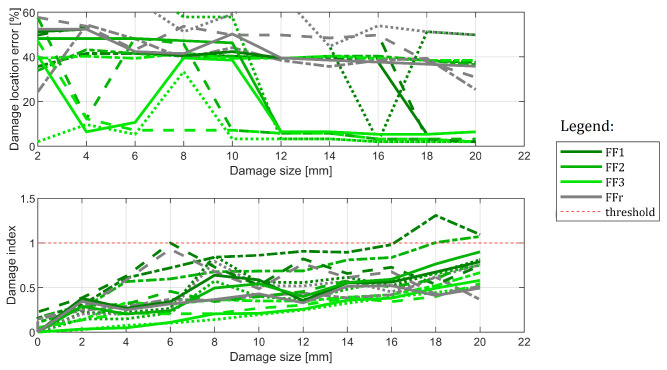
The results obtained for cases C#1, C#2, C#7 and C#8, that is for all the cases in which damage was located in the horizontal beam. The cases are denoted with dot-dash, dashed, solid and dotted lines, respectively.

Finally, cases C#1 and C#3 are considered. The results are provided in Figure 20. In this example, the results are much worse. While horizontal beam damage tends to influence damage indices and results in clear growth, this is not enough to trigger detection for two out of four methods. What is more, the location is erroneous and located somewhere in the middle of the structure. This shows that, while the methods react to damage, the provided size was not enough to cause reliable damage detection. It is not always the case, however. In Figure 21 the results for all 4 cases containing horizontal beam damage are shown (namely: C#1, C#2, C#7, and C#8). While only three damage indications remain in total (only three cases of threshold breach), the methods’ location capabilities tend to be better, with 8 out of 16 tests resulting in good damage location in the end. It is worth noting that FFr method did not manage to localize damage correctly even once. Thus, FF1 to FF3 methods were location-efficient for the biggest damage in 67% of cases.

### 6.4. Results Aggregation

**Figure 22:**
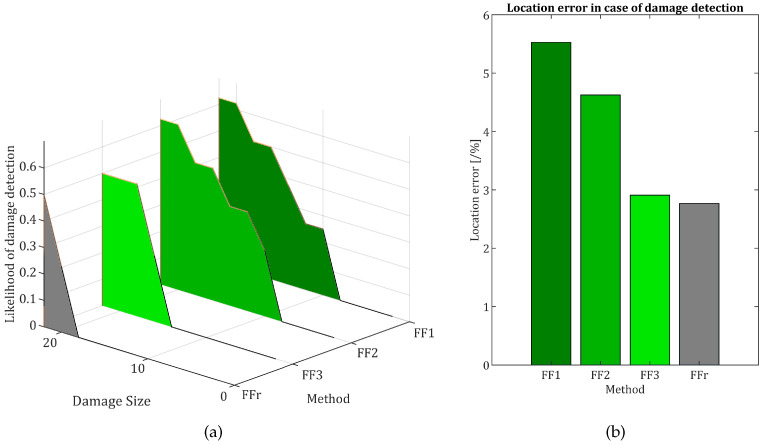
The results aggregated for all four methods under ivestigation: (**a**) damage location capabilities and damage detection capabilities in function of damage size and (**b**) damage location accuracy in the event of damage detection.

The aggregated results are presented for all the methods in Figure 22. As anticipated, out of the three Pareto front results, the first one (namely: FF1) obtained the highest error while being at the same time the most sensitive one, in 50% of cases detecting a 14 mm damage. The FF3 has the smallest location error out of the three methods, at the cost of being the least sensitive one, with detection of damage on average at 16 mm size. While being relatively precise in terms of damage location once it is detected, the reference configuration is at the same time least sensitive to damage, and on average, it can detect damage with 50% accuracy only in its final size. Thus, there is a clear trade-off: the methods that detect damage earlier tend to make bigger location errors. It is worth noting that all the results were presented for the whole set of results, including a problematic vertical beam. It is also worth noting that the threshold was not optimized for this particular experiment but was instead derived from the previous article’s data. Given the similar setup for metaparameters optimization, the solutions would achieve much better performance. This claim is supported by the fact that there is almost no noise in DI values, and they stay well below the threshold for 0 damage size, so there is much room for improvement here. One additional remark should also be given regarding the loading applied to the evaluated structure. The method’s sensitivity is in fact indirectly related to damage severity—as it depends on the deflection difference, which is dependant to both the load and the damage severity. This means that, for the same structure, more severe damage is more likely to be detected, but, also for the same structure, the higher loading of the structure cause the same severity of damage to be detected easier.

## 7. Summary and Conclusions

In this article, a new approach to deflection-based damage detection was introduced. Its primary contribution relies on introducing the AMS method for morphing data from any structure fixed at the end into a form similar to beam-like data. Therefore, it enables the usage of methods aimed at damage detection for beams. Five different damage detection methods were tested in the article: two of them (namely: SD and WV) are based on local deformation, two of them (namely: LD and LS) are based on general deflection shape and one fusion method that can incorporate in various extents input from all these sub-methods. Even though the fusion method was optimized based on historical data obtained for a different, beam-like structure, it was still able to detect damage in crane structure in most cases. The fusion method was compared with the previously reported algorithm and was proven to be much more efficient. While the method was proven to be generally successful and accurate, some problems have arisen regarding detecting damage in a horizontal part of a beam. While both the numerical simulation and some practical results suggest that the approach is efficient and could be of use, many cases were unsuccessful due to the relatively low signal-to-noise ratio. Therefore, the authors conclude that further work is required to determine the method’s efficiency for complex geometries.

## Figures and Tables

**Table 1:** EA configuration.

Parameter Name	Parameter Value
Population size	50
Generations	50
Initial mutation step	10% of draw range for each parameter
Mutation step decrease	Linear from Initial value to 10% of the initial value, reset after
	each 10 generations
Succession method	Pareto front, but no more than 50% of population
Elite succession	Yes, all individuals on front
Diversity control	No
Crossover	No

**Table 2:** Results of optimization for the Monit-POD dataset.

No	Detection Error [%]	Location Error [%]	SDs	SDw	LSs	LSw	LDs	LDw	WVs	WVw
FF1	11.20	6.7	67.80	37.61	6.58	26.81	36.63	154.56	28.09	0.00
FF2	14.94	4.5	71.25	16.82	45.21	68.52	76.40	169.38	17.06	1.51
FF3	24.48	3.3	19.04	1.96	2.08	31.02	16.34	116.24	25.93	0.00
FFr	24.48	3.3	35	40	350	40	0	0	0	0.00

**Table 3:** Experiment organization.

Case Number	Camera Number	Damage Location	Specimen Number
C#1	1	1	1
C#2	2	1	1
C#3	1	2	1
C#4	2	2	1
C#5	1	2	2
C#6	2	2	2
C#7	1	1	2
C#8	2	1	2

## Data Availability

Data used in this study is available on-demand from the corresponding author.
